# Method Verification of the Caretium XC-A30 Automated Erythrocyte Sedimentation Rate Analyser for Erythrocyte Sedimentation Rate

**DOI:** 10.21315/mjms2022.29.5.5

**Published:** 2022-10-28

**Authors:** Soemwit Khongwichit, Menapha Saelim, Yanisa Na-Songkhla, Hansuk Buncherd, Chawadee Nopparatana, Kanitta Srinoun

**Affiliations:** 1 Faculty of Medical Technology, Prince of Songkla University, Songkhla, Thailand; 2 Department of Pathology, Faculty of Medicine, Prince of Songkla University, Songkhla, Thailand

**Keywords:** erythrocyte sedimentation rate, automated ESR analyser, method verification

## Abstract

**Background:**

The erythrocyte sedimentation rate (ESR) analyser is widely used in haematological testing. In addition to the Westergren method, new automatic methods for ESR measurements have been developed. We aimed to study the reliability, precision, accuracy and stability of the Caretium XC-A30 automated ESR analyser.

**Methods:**

Ethylenediamine tetraacetic acid (EDTA)-treated blood samples were analysed via the Caretium XC-A30 automated ESR analyser and the Westergren method to compare accuracy. Precision was assessed using control samples and patient samples were classified into three groups—low, medium and high—according to their rates of sedimentation. Moreover, a stability test was performed.

**Results:**

The correlation coefficient of the results of the Caretium XC-A30 and Westergren analyses was 0.97. The correlation coefficient of ESR values obtained from the two methods assessed in the low, medium and high groups were r = 0.80, *r* = 0.68 and *r* = 0.74, respectively. The coefficient of variation of within-run (%CVw) and between-run (%CVb), with replicates performed with commercial controls samples, were 7.54% and 8.04% for the normal control and 4.68% and 3.50% for abnormal control, respectively. The %CVw obtained with patient samples in the low, medium and high groups were 10.68%, 13.13% and 4.45%, respectively. The Caretium XC-A30 measurements were stable for up to 24 h when samples were stored at 4 °C.

**Conclusion:**

The Caretium XC-A30 ESR analyser proved to be a suitable instrument for routine analysis of ESR.

## Introduction

The erythrocyte sedimentation rate (ESR) test is one of the most commonly presented haematology laboratory tests (1–4). The process of obtaining the ESR is described as three steps consisting of red blood cell (RBC) aggregation into rouleaux formations, followed by their precipitation, sedimentation and erythrocyte packaging (5, 6). Although ESR is usually used to investigate the acute phase response in inflammation (7, 8), its measurement is commonly affected by RBC shape, RBC size, RBC number, haematocrit and plasma protein concentration and especially by temperature and fibrinogen (6, 7). Currently, more specific inflammation testing has been reported; however, ESR remains useful in the diagnosis and follow-up of clinical conditions, such as rheumatoid arthritis (6, 7, 9) and Hodgkin lymphoma (10).

The standard method for ESR measurement is the Westergren method, as recommended by the International Council for Standardisation in Haematology (ICSH) (11, 12). This method uses a dilution of four volumes of blood to one volume of sodium citrate (13) and measures the distance erythrocytes fall after 1 h in mm (12, 14). However, the Westergren method has several well-described limitations for routine laboratory practice, including blood volume requirements and lengthy analysis time (> 1 h) (7, 15–17). To address these issues, a number of novel, modified semi-automated and alternate methods for ESR detection have been developed. Alternate ESR methods employ different principles than those of the Westergren method, such as photometric aggregometry or centrifugation (12) and these methods include ESR STAT PLUS (HemaTechnologies, Lebanon, NJ) (18), iSED (Alcor Scientific Inc., Smithfield, RI) or Test 1 (Alifax S.p.A., Polverara, Italy) (22–25). These methods perform the rouleaux formation step, the initial stage of ESR testing, resulting in a reduction in analysis time. Several automations, established by the conventional Westergren method, were introduced by using whole blood diluted with citrate, such as the StaRRsed (Mechatronics, Zwaag, the Netherlands) analyser (20, 26), Sediplast ESR (Polymedco, Cortlandt Manor, NY) (27) or the SEDIsystem (Becton Dickinson, Meylan Cedex, France) (28). However, the results obtained via these different methods can differ from the standard Westergren method (1, 12).

Caretium XC-A30 (Caretium Medical Instruments, Shenzhen, China) is a newly developed, alternate automated ESR analyser. This ESR analyser was designed to improve the laboratory workflow of ESR measurement, use smaller blood volumes and utilise infrared photometric aggregometry.

The purpose of our study was to investigate the analytical performance of this new device, the Caretium XC-A30 automation analyser and compare the results with those obtained from the Westergren method.

## Materials and Methods

### Patient Samples

Blood samples from 162 standard hospitals were collected in ethylenediamine tetraacetic acid (EDTA) tubes from whom ESRs were requested at Songklanagarind Hospital in September 2020–October 2020. EDTA-anticoagulated blood samples aliquoted into 1.6 mL into sodium citrate tubes, were used for ESR measurements by the Westergren method and 1.28 mL aliquots prepared in IMPROVACUTER^®^ESR tubes (Improve Medical, Guangzhou, China) were used for ESR measurements in the Caretium XC-A30 ESR analyser. Haemolysed and clotted samples were excluded. Blood samples were examined within 4 h, after collection according to the ICSH guidelines (8, 11).

### The Westergren Method

The conventional procedure for the Westergren method was performed by diluting four volumes of blood with one volume of sodium citrate according to the ICSH protocol (11). Samples were subsequently aspirated into the Westergren pipette at 300 mm length and mounted vertically in a Westergren rack without vibration. The distance of sedimentation was assessed visually after 60 min and then reported in mm (14).

### Caretium XC-A30 Automated ESR Analyser

Caretium XC-A30 is an automated analyser that measures ESR using infrared photometry. The patients’ whole blood samples were drawn into IMPROVACUTER^®^ ESR tubes that contained sodium citrate (3.2%) as the anticoagulant. In this way, citrate diluted blood (four volumes of blood to one volume of citrate) was achieved. The samples were mixed at least five times. To minimise the turnaround time, the sedimentation was measured by using an infrared optical sensor after 30 min. The results are then given in mm and were automatically standardised into 60 min measurement times using values obtained at 18 °C according to the manufacturer.

### Precision Study

Within-run and between-run precision were determined by analysing normal and abnormal ESR ranges obtained using a commercially available control, the Liquichek™ Sedimentation Rate Control (Bio-Rad Laboratories, Inc.), which is composed of stabilized human whole blood. Using the Clinical Laboratory Standards Institute (CLSI) protocol (29, 30), within-run precision was assessed by performing 20 consecutive measurements. Between-run precision was analysed by processing the manufacturer’s control material three times daily for 20 days. Additionally, within-run precision was assessed by performing 20 replicate measurements of three patient samples in each of the low (< 20 mm), middle (21 mm–80 mm) and high (> 80 mm) ESR values groups. Means, standard deviations and coefficient of variations (CVs) were calculated. Imprecision was calculated as the CVs of within-run (%CV_w_) and between-run (%CV_b_) precision. %CV_w_ and %CV_b_ were used for the calculation of total imprecision (CV_t_) using the following equation:


$${\text{CV}}_{t}=\sqrt{{\left({\text{CV}}_{\text{w}}\right)}^{2}+{\left({\text{CV}}_{b}\right)}^{2}}$$

### Method Comparisons Study

A total of 125 samples were chosen randomly and then used for method comparison. Samples were investigated in parallel by the Westergren and Caretium XC-A30 automated methods (31). Passing-Bablok linear regression analysis was used to determine the degree of correlation between the results of the Westergren methods and those of the Caretium XC-A30 automated ESR analyser and Bland-Altman difference plots were used to assess absolute differences. Correlation coefficients and biases for samples in the low (< 20 mm), middle (20 mm–60 mm) and upper third (> 60 mm) of the analytical range were determined (12, 14). To compare the two methods, data distribution was assessed by the Shapiro-Wilk test. Spearman’s rank correlations (*r*) were used to compare results between manual methods and the automated ESR analyser. Statistical significance was assumed to be *P* < 0.05. The MedCalc software free trial version was used in the evaluations.

### Sample Stability

Sample stability analysis was performed randomly on 22 samples. The samples were then divided into aliquots and stored at either room temperature (RT) or 4 °C. Samples stored at 4 °C were then allowed to return to room temperature before re-testing. ESR measurements were performed by the Caretium XC-A30 automated ESR analyser at 4 h, 6 h, 8 h and 24 h after collection. The results were compared using a parametric paired *t*-test. Values of *P* < 0.05 were accepted as statistically significant (SPSS 23.0, SPSS Inc., Chicago, Ill., USA).

## Results

### Method Comparisons Study

**Figure 1:**
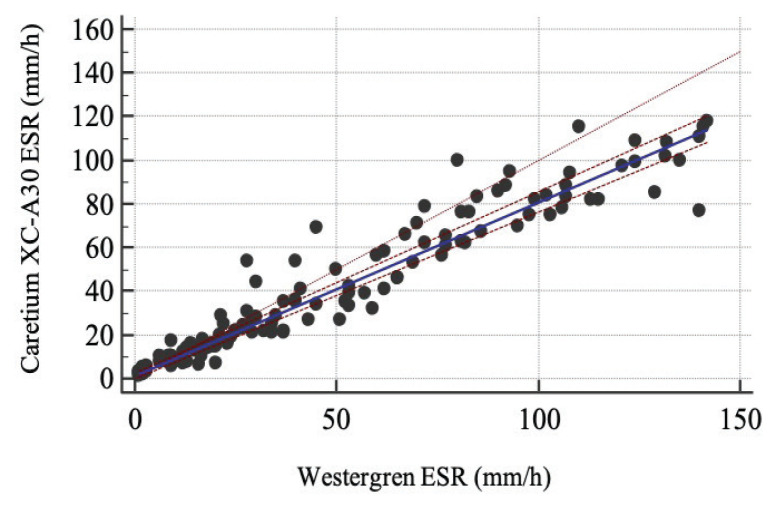
Correlation of the Caretium XC-A30 analyser and the Westergren methods

**Figure 2:**
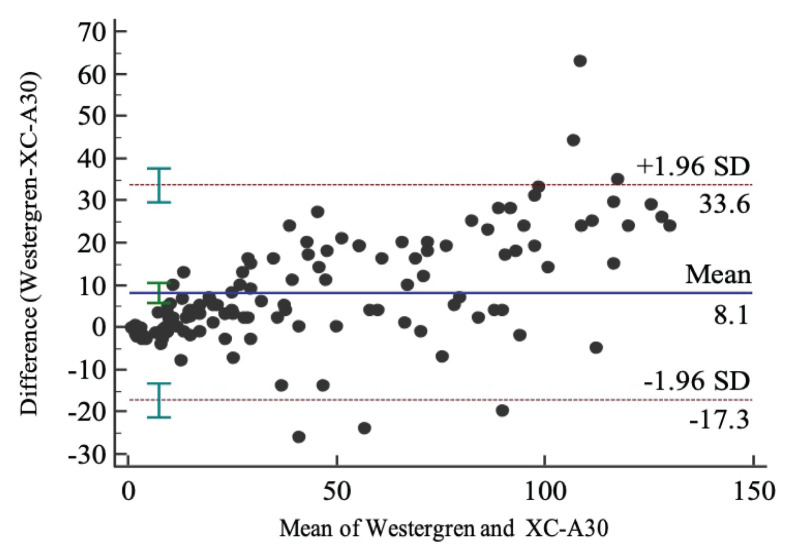
Bland-Altman plots of the difference comparing the Caretium XC-A30 analysers and the Westergren methods

The ESR results measured by using the Caretium XC-A30 analysers were compared with the standard Westergren methods. The obtained Spearman’s rank correlation (*r*) was 0.97 (95% confidence interval [CI]: 0.96, 0.98; *P* < 0.0001). Passing-Bablok linear regression showed a regression equation *y* = 1.15 + 0.80*x*, *y*-intercept of 1.15 (95% CI: 0.05, 2.44) and slope of 0.80 (95% CI: 0.76, 0.83) (Figure 1). Bland-Altman difference plot analysis revealed a positive mean bias of 8.13 (95% CI: 5.83, 10.43) (Figure 2). We classified the results into subgroups as follows: low (< 20 mm), middle (20 mm–60 mm) and upper third (> 60 mm). The results of the Passing-Bablok linear regression and Bland-Altman difference plot analyses are shown in Table 1. Measurements obtained by the methods from samples in the lower third of the analytical range presented a good correlation across the two methods (*r* = 0.80; *P* < 0.0001), but a moderate correlation was found with samples from the middle and upper third of the analytical range (*r* = 0.68; *P* < 0.0001 and *r* = 0.74; *P < 0.0001*, respectively). The Bland-Altman difference plot exhibited a significant increase in the differences between the two tests at ESR values > 60 mm with an observed mean difference of 16.4 mm (95% CI: 11.18, 21.70).

### Precision

The CV values for the within-run and between-run precision analysis were 7.54% and 8.04% for the normal control and, 4.68% and 3.50% for the abnormal control, respectively. The CV values for the total precisions of the normal and abnormal commercial controls were 11.02% and 5.84%, respectively (Table 2). The CV values for the within-run precision analyses of patient samples at low, medium, and high were 10.68%, 13.13%, and 4.45%, respectively (Table 3).

### Sample Stability Test

The results of the stability studies of the Caretium XC-A30 automated ESR analyser are shown in Table 4. The specimens were stored at 4 °C. The ESR results obtained using samples stored at 4 °C did not change significantly after 24 h of collection; whereas, the ESR results of the samples at room temperature diminished significantly at 24 h.

## Discussion

The ESR test is a commonly used laboratory test for evaluating acute phase response inflammation (8). Despite the Westergren method being the gold standard method for the measurement of ESR, this method is time-consuming, requires a large volume of blood samples, and is laborious (7, 15–17). To overcome these issues, several automated systems are now available for ESR measurement (12). According to the ICSH classification guidelines, these novel technologies are characterised as modified Westergren methods, when they feature some modifications to the Westergren methodology and alternate ESR methods for those created using different methodological principles, such as centrifugation or photometric rheology (20). Caretium XC-A30 is a newly established, automated alternate ESR analyser. It is a small bench-top equipment (40 cm × 30 cm × 20 cm) that has been established for small to medium-sized laboratories. This method is based on infrared photometric aggregometry, which produces results in 30 min resulting in a reduction in analysis time. Caretium XC-A30 has also been designed to use less blood volume. To our knowledge, this study is the first to report that Caretium XC-A30 shows satisfactory precision characteristics and comparability with the referenced standard Westergren method.

Herein, we demonstrated good precision of Caretium XC-A30 with commercial control samples at normal evaluated at normal levels than in modified Westergren ESR automation, StaRRsed (20) and Ves-Matic Cube 200 (32) but compared to evaluations obtained from other methods, evaluation using abnormal control levels resulted in a slight increase in imprecision. Based on similar determination techniques, the imprecision of Caretium XC-A30 with commercial control samples evaluated at both normal and abnormal levels was higher than those obtained with iSED (19) and Test-1 analyser (25).

According to CLSI H2-A4 guidelines, acceptable performance limits are described for ESR results and CV (%) values between 10.88 and 38.88, for different ESR values are considered acceptable performance limits (8). In our study, we assessed patient samples using low, medium and high ESR values, with 20 replicate measurements. The within-run precisions were 10.68%, 13.13% and 4.45% in the low, medium and high ESR groups, respectively. Caretium XC-A30 seems to have precisions within acceptable performance limits described in the guidelines. The evaluated patient sample ESR levels were similar to already reported data for iSED and Ves-Mastic cube 200, with higher CVs observed at low and medium ESR levels (19–21). Interestingly, our data demonstrated lower within-run decreasing CVs at the low and high ESR levels than those described in previously reported data (19–21, 25).

Our study comparing the performance of the Caretium XC-A30 and the Westergren method revealed a good correlation. The overall correlation coefficient was 0.97 (95% CI: 0.96, 0.98; *P* < 0.0001). A significant mean difference of 8.13 (95% CI: 5.83, 10.43) was detected in this study. In the subgroup analysis, there was a good correlation between Caretium XC-A30 and the Westergren method at ESR levels in a lower analytical range. A moderate correlation of the two analysers was found using samples evaluated at the middle and upper third of the analytical range. However, the measurements obtained from upper third of the analytical range showed a large mean bias of 16.4 mm (95% CI: 11.18, 21.70).

The accuracy of the overall correlation coefficient of our group was similar to that reported in the modified Westergren ESR principles, such as the SEDI system (7, 28) and StaRRsed (7). However, our result showed a higher correlation coefficient than those obtained with the Ves-Matic Cube 200 (7, 25, 32). The overall correlation coefficient was similar to those previously reported using the iSED (19, 20) and Test-1 analyser (25), of which are each alternate ESR measurement methods. Unlike the other methods, the results of subgroup analysis for ESR values obtained using the Caretium XC-A30 method have been reported to have a good or moderate correlation with the ESR values obtained from the standard Westergren methods. This result is even more obvious when evaluating the correlation coefficients per each ESR level group, including those within the upper third of the analytical range (18–22, 32). However, the ESR values within this higher analytical range obtained from the Caretium XC-A30 were significantly higher than those of the Westergren method, with a mean difference of 16.4 mm (95% CI: 11.18, 21.70), meaning that a patient could have high ESR if detected by Caretium XC-A30 and normal ESR if detected with the Westergren method, or vice versa, which could each possibly cause different clinical interpretations. These inconsistencies can be attributed to the principal differences of the two techniques and the difference in the timing of ESR measurements, as Caretium XC-A30 estimates ESR by kinetic assessment of rouleaux formation in the initial sedimentation phase, while the classical Westergren method measures ESR after all three phases of sedimentation.

A major limitation of Westergren ESR methods is the requirement to perform the test within 4 h from the time of sample collection when stored at room temperature (12). Caretium XC-A30 ESR results, when the specimens were stored in a refrigerator, did not change significantly after 24 h of collection but ESR results of room temperature samples declined significantly at 24 h. The reduction in sample ESR after 24 h has been described by other reports. They demonstrated that this reduction could depend on RBC swelling and the reduction in sialic acid within the RBC cell membranes (5, 33). Our results were similar to stability testing conducted in the Ves-Matic Cube 200 ESR experiment; (21, 25, 34), wherein the results obtained using the iSED and Test-1 analyser were stable at room temperature and 4 °C after 24 h of collection (20, 25).

In summary, the Caretium XC-A30 analyser provides accurate ESR measurements and demonstrates acceptable concordance with the gold standard Westergren method. However, the limitation of this accuracy study is that the sample size may not sufficiently reflect the results of all pathological ESR levels examined by this instrument. Hence, the need for subsequent studies with a larger number of samples should be conducted in order to reveal better accuracy. Additionally, the effects of interfering elements such as fibrinogen and paraprotein, which increase rouleaux formation, were not assessed in the study.

## Conclusion

In conclusion, the Caretium XC-A30 analyser is a novel alternative ESR method providing rapid measuring time and precise and accurate determination of the ESR. The Caretium XC-A30 analyser offers advantages, including reliability, reduced sample volumes requirements and extended sample stability. However, at the upper third of the analytical range, the large mean bias difference between the Caretium XC-A30 analyser and Westergren method was remarkable; thus, the results should be interpreted with caution. Further studies assessing the impact of alternative ESR methods on the clinical investigation should be undertaken.

## Figures and Tables

**Table 1:** Comparison statistics at the lower, middle, and upper third of the analytical range

Analytical range	*N*	Bias (95% CI)	Correlation coefficient (*r*)	Intercept	Slope
ESR (< 20 mm)	44	1.35 (0.14, 2.57)	0.80	2.83 (0.96–3.75)	0.67 (0.58–0.79)
ESR (20 mm–60 mm)	41	7.3 (3.83, 10.78)	0.68	4.23 (−3.31–9.56)	0.71 (0.56–0.92)
ESR (> 60 mm)	40	16.4 (11.18, 21.70)	0.74	16.33 (1.43–34.5)	0.67 (0.50–0.81)

**Table 2:** Within-run and between-run precision analysis with commercial controls

	Mean ± SD (mm)	CV (%)	Range (mm)
Normal control (lot 27841)			
Within-run precision	4.39 ± 0.33	7.54	3.91–5.92
Between-run precision	5.19 ± 0.42	8.04	4.43–5.93
Total precision		11.02	
Abnormal control (lot 27841)			
Within-run precision	44.86 ± 2.10	4.68	41.47–57.83
Between-run precision	50.50 ± 1.77	3.50	41.17–53.67
Total precision		5.84	

**Table 3:** Within-run precision analysis with patient samples

	Mean (SD) (mm)	CV (%)	Range (mm)
Low	8.81 (0.98)	10.68	0–20
Medium	33.40 (5.15)	13.13	21–80
High	87.81 (3.88)	4.45	> 80

**Table 4:** Evaluation of stability study

	Fresh (*n* = 22)	4 h (*n* = 22)	6 h (*n* = 22)	8 h (*n* = 22)	24 h (*n* = 22)
Samples stored at RT Mean (SD) (mm)	8.98 (5.25)	8.66 (4.22)	9.18 (4.22)	8.68 (5.20)	4.83 (1.97)
Mean of differences (mm)		0.32	−0.19	0.30	4.15
95% CI		−2.1, 2.75	−5.19, 1.21	−2.36, 2.96	2.11, 6.20
*P*-value		0.792	0.873	0.500	0.000^a^
Samples stored at 4 ^0^C Mean (SD) (mm)	8.62 (5.15)	10.47 (5.83)	9.85 (5.95)	9.95 (6.13)	10.19 (5.81)
Mean of differences (mm)		−1.85	−1.23	−1.33	−1.57
95% CI		−4.64, 0.94	−4.06, 1.59	−4.21, 1.55	−4.36, 1.22
*P*-value		0.191	0.386	0.358	0.266

Notes: ESR values are expressed as mean (SD);

a There is a significant difference (*P* < 0.05) from the fresh ESR result
